# Work-Related Predictors of Sleep Quality in Chinese Nurses: Testing a Path Analysis Model

**DOI:** 10.1097/jnr.0000000000000319

**Published:** 2019-09-20

**Authors:** Yuan LI, Jinbo FANG, Chunfen ZHOU

**Affiliations:** 1 Doctoral Candidate, RN, Junior Nurse, West China School of Nursing, Sichuan University, Chengdu City, Sichuan Province, China; 2 PhD, RN, Associate Professor, Nursing Department, West China Hospital, Sichuan University, Chengdu City, Sichuan Province, China; 3 MSN, RN, Junior Nurse, Mental Health Center of West China Hospital, Sichuan University, Chengdu City, Sichuan Province, China.

**Keywords:** Chinese nurses, path analysis, predicting model, sleep quality, work-related factor

## Abstract

**Background:**

Good sleep is essential to human health. Insufficient quality sleep may compromise the wellness of nurses and even jeopardize the safety of patients. Although the contributors of sleep quality in nurses have been previously studied, the direct and indirect effects of modifiable work-related predictors remain uncertain.

**Purpose:**

The study was designed to explore the direct and indirect effects of modifiable work-related factors on sleep quality in Chinese nurses.

**Methods:**

A multistage sampling method was employed in this cross-sectional study to recruit 923 participants. An evidence-based predicting model was postulated and then subsequently tested and optimized using path analysis.

**Results:**

The final model fit the data well, with the involved predictors accounting for 34.1% of the variance in sleep quality of the participants. Shift work, job demands, exposure to hazards in work environments, chronic fatigue, and inter-shift recovery were identified as direct predictors, while whereas job satisfaction, job control, support at work, and acute fatigue were identified as indirect predictors.

**Conclusions/Implications for Practice:**

Sleep quality in Chinese nurses is influenced directly and indirectly by various modifiable work-related factors. Interventions such as adjusting work shifts and reducing job burdens should be prioritized by administrative staff to ensure the sleep quality and clinical performance of Chinese nurses and to subsequently improve nursing care quality.

## Introduction

Although “sleep quality” is widely used, this term lacks definitional consensus. A panel of experts that was recently assembled to address this intellectual enquiry drew the conclusion that sleep continuity variables such as sleep latency, length of sleep, and frequency of awakenings as well as sleep efficiency are appropriate indicators of good sleep quality across the human life-span (Ohayon et al., 2017). Adequate quality sleep and feeling well-rested are required to remain healthy and productive. It has been reported that sleep deprivation over the short term negatively impacts mood, vigilance, and rapid response capabilities, whereas sleep deprivation over the long term leads to thought retardation (Han, Yuan, Zhang, & Fu, 2016), chronic fatigue (Fang, Qiu, Xu, & You, 2013), elevated mortality, cardiovascular diseases, and obesity and diabetes (Itani, Jike, Watanabe, & Kaneita, 2017). In addition, sleep challenges may have detrimental psychological consequences such as anxiety and depression. Furthermore, the reciprocal correlation between sleep condition and psychological state has been noted (Kouros & El-Sheikh, 2015). Of particular interest is the finding of a strong U-shaped relationship between sleep duration in employees and socioeconomic loss, with the nadir of the “U” unveiling itself for the recommended 7–8 hours of sleep per night (Burton, Chen, Schultz, & Li, 2017). More frequent healthcare utilization, productivity loss, absenteeism, and presenteeism resulting from poor sleep may contribute to reduced economic outcomes.

The Centers for Diseases Control and Prevention have declared that “sleep is essential for good health; it is a vital necessity, not a luxury.” However, inherently involved in intense and hectic work, nurses are always at a high risk of insufficient sleep and sleep disorders (Kunzweiler et al., 2016). In an assessment using the Pittsburgh Sleep Quality Index (PSQI), over 70% of Chinese nurses in a prior study were reported to have experienced poor sleep quality, which is a ratio that is much higher than the 39.4% reported for the general population (Lin, Liao, Chen, & Fan, 2014). Sleep problems in nursing personnel may not only compromise well-being but also impair judgment and performance, which may jeopardize the safety of patients (Eanes, 2015). A recent study conducted to evaluate medical errors made by nurses found that approximately 40% of nurses had committed at least one medical error during their career and that 78.3% of nurses identified sleepiness as a main culprit of nurse medical error (Kahriman & Öztürk, 2016). As patient safety is a priority of healthcare industries, the widely acknowledged relationship between the sleep quality of nurses and patient safety requires more research into this issue. Over the past decade, individual factors, behavioral factors, physical factors, and psychosocial factors have been identified as contributors to poor sleep in nurses (Chung, Liu, Lee, & Hsu, 2013; Han et al., 2016). Nevertheless, most of these factors, for example, age and marital status, are not subject to immediate utilization in related organizational interventions. Although work-related factors are more readily manipulated by administrative staff, few studies have addressed these factors. In addition, most related research has focused on the effects of shift work or on isolated cases only (Caruso, 2014; Lin et al., 2014). Thus, it remains unclear how these proposed factors interrelate with each other and impact sleep quality. Therefore, this study conducted a multiaspect-oriented investigation of modifiable work-related factors that are associated with sleep quality in Chinese nurses and then proposed, tested, and optimized an innovative, evidence-based hypothesized model using path analysis.

**Figure 1.**
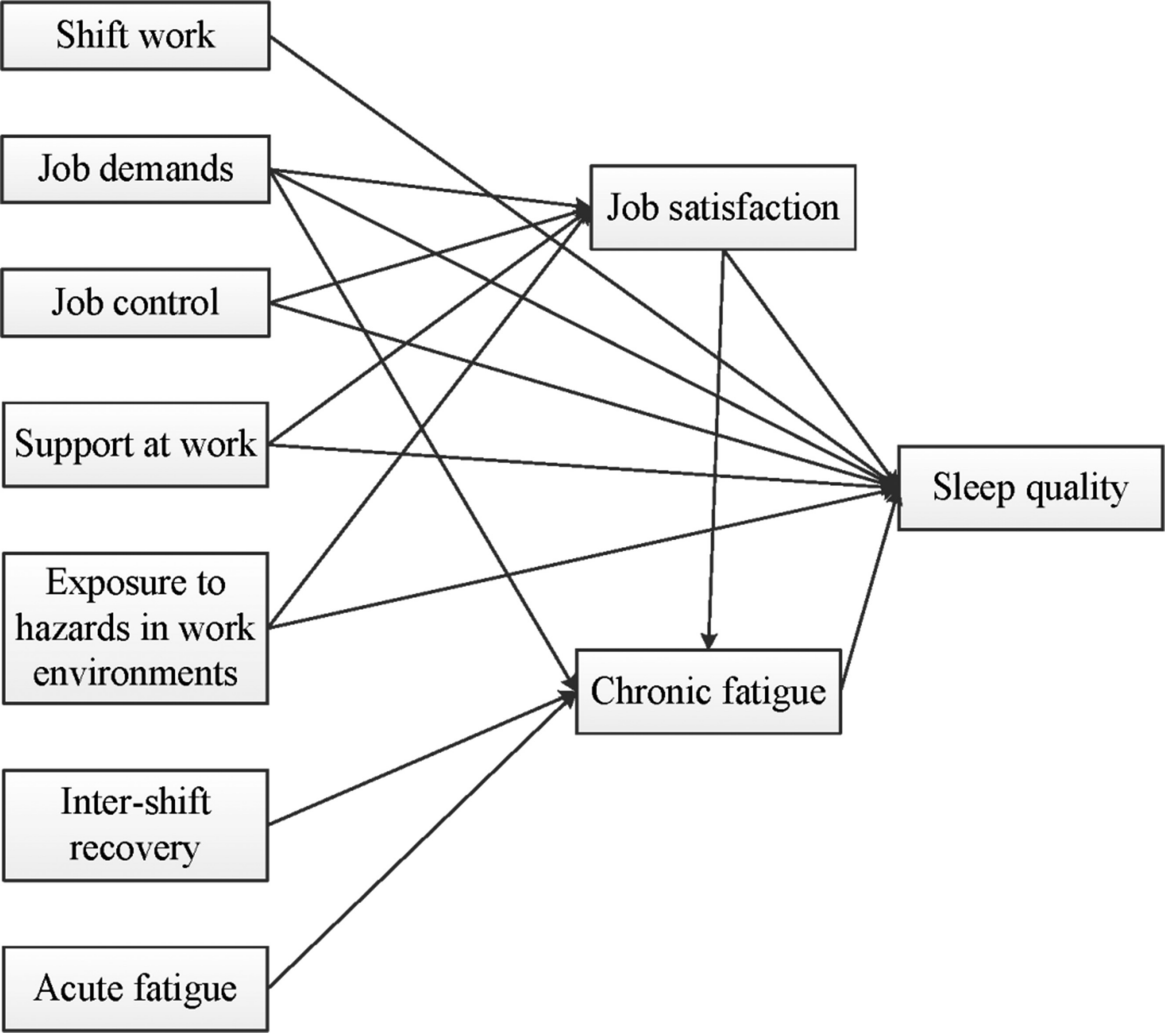
The original hypothesized model for predicting sleep quality in Chinese nurses.

After a thorough literature review, an initial model incorporating nine explanatory variables was established (Figure 1). Except for intershift recovery and acute fatigue, the other variables, including shift work, job demands, job control, support at work, exposure to hazards in work environments, job satisfaction, and chronic fatigue, were posited as direct factors. Direct factors are factors that act on the outcome (sleep quality) directly, rather than through other intermediate factors. Furthermore, job satisfaction and chronic fatigue were hypothesized as mediators of the developed model. Specifically, job demands, job control, support at work, and exposure to hazards in work environments were each perceived to affect job satisfaction independently (Zhang et al., 2014) and to act as mediators of sleep quality, whereas chronic fatigue played a mediating role between job demands, job satisfaction, intershift recovery, and acute fatigue and the outcome (Fang et al., 2013). As intershift recovery and acute fatigue were not found to impact the outcome directly, they were presumed to be indirect factors that affect sleep quality through the mediating factor of chronic fatigue.

Shift work was identified repeatedly as one of the significant factors affecting sleep quality in nurses (Lin et al., 2014). Because healthcare services in hospitals must be offered on a 24-hour basis, nursing work is usually arranged in rotation, making it impossible for nurses to rest on a regular schedule. Unfortunately, sleep times misaligned with the endogenous circadian rhythm may trigger difficulties in falling asleep, early awakenings, and disrupted sleep (Caruso, 2014). Recent evidence supports that nurses on night duty are more inclined than their day-shift colleagues to manifest inferior sleep quality (Han et al., 2016). Hence, shift work was assumed to be a direct predictor of sleep quality in this study.

A synthesis of job demands (mainly psychological), job control (decision latitude), and support at work (support available from coworkers and supervisors) has been shown to credibly predict job strain (Akerstedt, Nordin, Alfredsson, Westerholm, & Kecklund, 2012), which has been documented to relate inversely to sleep quality (Kunzweiler et al., 2016). A recent study of Nigerian nurses suggests that excessive job demands, if not buffered by job control and support, may give rise to mental exhaustion and ill health (van Doorn, van Ruysseveldt, van Dam, Mistiaen, & Nikolova, 2016). Additional studies verifying the links between these three occupational features and sleep quality confirm that high work demands are among the risk factors for sleep quality and that limited control over job and lack of support further elevate these risk factors (Akerstedt et al., 2012; Ruotsalainen, Verbeek, Mariné, & Serra, 2014). Therefore, this study hypothesized that job demands, job control, and support at work should associate directly with quality of sleep in nurses.

Hazardous work environments are presumed to be associated with an elevated risk of physical injury and a lowered degree of safety support, and the cumulative effects of prolonged exposure to these environments carry a higher risk of adverse impacts. A recent literature review (Caddick, Gregory, Arsintescu, & Flynn-Evans, 2018) on the environmental parameters of good sleep concluded that artificial light, ambient noise, ambient temperature, and air quality are crucial environmental factors affecting sleep deprivation. In addition, cross-sectional research on organic solvents and electromagnetic field exposures (Liu et al., 2014) ascertained that these factors affected sleep quality negatively in various study subgroups. Accordingly, exposure to hazards in work environments was incorporated into this study as a direct predictor of sleep quality.

Job satisfaction is a noteworthy construct, especially in the realm of administration, that may predict work behaviors such as organizational citizenship and withdrawal behaviors such as absenteeism and turnover. The effects of poor job satisfaction may be so far-reaching that the overall feelings from one's job experience may even affect everyday (non-job-related) life activities. As reported by Kunzweiler et al. (2016), sleep disruptions are closely related to poor job satisfaction and sleep quality decreases proportionally with job dissatisfaction. A multisite research in China found that nearly half of Chinese nurses were dissatisfied with their current jobs, that a supportive work environment (support at work) may play an alleviating role, that perceived high job demands (job demands) and limited job autonomy (job control) may intensify dissatisfaction, and that an unfavorable practice environment with continual security threat (exposure to hazards in work environments) may aggravate dissatisfaction even further (Zhang et al., 2014). Thus, this study assumed that job satisfaction both impacts sleep quality directly and mediates the relationships between job demands, job control, support at work, and exposure to hazards in work environments and quality of sleep in Chinese nurses.

Fatigue in nursing staffs is a long-standing problem that comprises both chronic (long-term) and acute (short-term) aspects. Numerous studies have validated the significant correlation between employee fatigue and sleep quality (Han et al., 2016; Korompeli, Chara, Chrysoula, & Sourtzi, 2013). Korompeli et al. (2013) revealed a positive connection between chronic fatigue and all sleep scales and summarized that the population of nurses with elevated chronic fatigue levels overlapped closely with those experiencing relatively severe sleep disturbance. Empirical studies have verified that chronic fatigue evolves from the persistent failure to recover from acute fatigue and that intershift recovery may help counteract the onset of this type of fatigue (Winwood, Bakker, & Winefield, 2007). A well-supported structural equation model (Fang et al., 2013) indicated that, in addition to acute fatigue and intershift recovery, chronic fatigue is also directly influenced by job demands and job satisfaction. In light of the abovementioned evidence, this study included chronic fatigue into the hypothesized model as a direct factor of the outcome and as a mediator between job demands, job satisfaction, intershift recovery, and acute fatigue and sleep quality.

The problem of sleep quality among nurses has become a critical issue for healthcare systems because of its high prevalence, negative effects on individual health and patient care quality, and associated costs. The modifiable work-related factors that impinge on sleep quality in nurses merit further exploration to evaluate and enhance the current situation. This study was designed to explore and describe the modifiable work-related factors that are capable of predicting sleep quality in Chinese nurses, to differentiate the direct and indirect effects of these identified factors, and to provide research-based evidence to help guide the future development of strategies to improve the sleep quality of nurses.

## Methods

### Sample and Setting

This cross-sectional design study was conducted in Chengdu, China. The inclusion criteria for participants were (a) working in inpatient departments, (b) providing direct patient care, and (c) working 8-hour/night shifts, whereas exclusion criteria were otherwise qualified nurses who were currently on maternity leave, had physical/mental diseases, or were taking medications during the data collection period. The sample size of the path analysis was determined using the formula: N = L/f^2^ + k + 1 (Cohen, Cohen, West, & Aiken, 2003), where N = number of sample, L = noncentrality parameter, f^2^ = effect size for regression statistics (calculated using f^2^ = R^2^/1-R^2^), and k = the number of predictors for multiple correlation testing. When Type I error = 0.05 and power = 0.8, the L value was found to be 15.65 (Cohen et al., 2003). According to previous studies (Chung et al., 2013; Kunzweiler et al., 2016) on sleep quality, the *R*^2^ ranges from .130^2^ to .785^2^. To maximize the statistical power, .130^2^ was adopted to calculate the effect size (f^2^). Taking the number of variables and nonresponse rate into consideration, 1,071 nurses were invited to participate in this study.

A multistage sampling process was implemented to recruit participants. First, one general hospital was selected from each of the five districts in Chengdu by simple, random sampling. Second, we collected the lists of all eligible nurses from the selected hospitals and randomly sequenced each nurse by computer. Then, the nurses who would be invited to participate were selected from this list using systematic sampling.

### Data Collection

Ethical approval was obtained before commencing the study from the Institutional Review Board for Clinical Trials and Biomedical Research at the West China Hospital, Sichuan University (No. 2008SCU086). Official permission was granted by the targeted nursing departments, and written informed consent was obtained from all of the participants. Permissions from the original authors who had developed the scales that were used in this study were obtained by e-mail. The questionnaires used in this study contained no identifiable information. Data were analyzed at an aggregated level, and the authors were not able to identify the data of individual participants during or after data collection. Questionnaires were distributed by trained staff from each nursing department between December 2012 and April 2013. Upon distributing, every participant was informed about the study objectives and given detailed instructions. They were required to take his or her own time to complete and then seal the questionnaire and then to submit the sealed questionnaire within 3 days to the box outside their wards. One thousand nineteen completed questionnaires were returned, of which 923 were considered valid, giving an overall effective response rate of 86.18% (923/1,071).

### Instruments

#### Demographic form

Demographics including age, marital status, education, professional and position title, years of working as a nurse, working unit, and number of night/evening shifts per month were collected.

#### Pittsburgh Sleep Quality Index

The PSQI is a psychometrically sound instrument that is widely used to quantify the subjective sleep quality of respondents over the immediately preceding 1-month period. The Chinese version, validated by Tsai et al. (2005), was applied here to estimate sleep quality in Chinese nurses. Seven components comprising 19 items constitute this self-report questionnaire. The component scores are summed to obtain a single, global score, which has a total possible range of 0–21, with higher points indicating worse sleep condition. According to Tsai et al., using 5 as the cutoff point for poor sleep gives the instrument a sensitivity and a specificity of 98% and 55%, respectively, and the Chinese PSQI showed an adequate reliability coefficient of .83. In this study, the reliability test for the Chinese PSQI yielded a Cronbach's alpha of .73.

#### Job Content Questionnaire

The Job Content Questionnaire, developed by Karasek (1985), was intended to measure the psychosocial characteristics of jobs. The verified Chinese version was adopted in our study. The Job Demands Subscale, Job Control Subscale, and Support at Work Subscale are the core components of the Job Content Questionnaire, which are each assessed using a 4-point Likert scale, with 1 indicating “*strongly disagree*” and 4 indicating “*strongly agree*.” In this study, the Cronbach's alpha for the three subscales were .76, .68, and .90, respectively.

#### Exposure to Hazards in Hospital Work Environments scale

The Exposure to Hazards in Hospital Work Environments scale is our modified Chinese version of the Hospital Occupational Hazards Scale (Gillmore, 1990), which was constructed to assess how frequent nurses in hospital work environments are exposed to physical, chemical, and biological hazards. This 20-item tool is scored using a Likert-type 5-point scale (1 = *never*, 5 = *always*). This study presented good internal consistency (Cronbach's alpha = .82) and test–retest reliability (.76 within a 2-week period), and the content validity index was rated at .87.

#### Job Satisfaction Scale

The Job Satisfaction Scale (Karasek, 1985) was designed to evaluate overall feelings about one's job. The verified Chinese version (Cheng, Luh, & Guo, 2003) with five self-report items was employed in this study. The weighted item scores add up to a total score that ranges from 0 to100, with higher scores representing higher levels of job satisfaction (Karasek, 1985). In this study, the Cronbach's alpha was .76 for this instrument.

#### Occupational Fatigue Exhaustion Recovery scale

The Occupational Fatigue Exhaustion Recovery scale (Winwood, Lushington, & Winefield, 2006), originally developed based on nursing populations, is the only instrument that is currently able to differentiate between chronic and acute fatigue states and to measure effective fatigue recovery between shifts. The Chronic Fatigue Subscale and Acute Fatigue Subscale measure the subjective feeling of fatigue, whereas the Intershift Recovery Subscale measures the feeling of recovery between work shifts. The English questionnaire was translated into Chinese and then back-translated into English by two bilingual experts until complete consent was achieved. The accuracy and consistency of meaning were scrutinized by a nursing professor. Occupational Fatigue Exhaustion Recovery items are scored on a 7-point Likert scale (0 = *strongly disagree*, 6 = *strongly agree*). The Cronbach's alphas were .81 and .82 for the Chronic and Acute Fatigue Subscales, respectively, and .86 for the Intershift Recovery Subscale.

### Statistical Analysis

Data were analyzed using SPSS Version 23.0 and Amos Version 23.0 (IBM Corp., Armonk, NY, USA). Considering the different scales used by each instrument, all of the data, with the exception of demographic information, were transformed into a 0–100 ranking scale. Descriptive analyses were then used to delineate the sample characteristics. Pearson correlation analyses were first conducted to test if the associations among the variables met the prerequisites for performing path analysis. Next, path analysis was applied to test and modify the model. The model-data fit was evaluated using χ^2^, χ^2^/*df*, root mean square error of approximation (RMSEA), normed fit index (NFI), comparative fit index (CFI), and goodness of fit index (GFI). A nonsignificant χ^2^ and χ^2^/*df* remaining in between 1 and 2 indicate an acceptable model fit, and the RMSEA should be less than .05. With regard to NFI, CFI, and GFI, values no less than .90 indicate a good model fit, whereas values above .95 indicate an excellent fit (Cohen et al., 2003). The bootstrap resampling technique, especially appropriate for nonnormal data, was employed in this study to test the significance of direct and indirect variable effects (MacKinnon, Lockwood, & Williams, 2004). Typically, the resampling process should be repeated at least 1,000 times during analysis as a means of mimicking the original sampling process to generate a more reliable confidence interval (MacKinnon et al., 2004). This study set 1,000-times bootstrapping as recommended to yield a 95% confidence interval. Moreover, the squared multiple regression correlation coefficient (*R*^2^) was estimated to identify the variance in sleep quality that was explained by proposed factors.

## Results

### Sample Characteristics

The participants had a mean age of 29.73 years; most were married (62.3%); 70.6% held an associate degree, and 17.5% held a baccalaureate or higher degree; nearly half were junior nurses (49.2%); and the overwhelming majority (92.6%) were staff nurses. The median year of professional nursing experience was 8.00. Nearly half (44.6%) of the participants worked more than eight night/evening shifts per month, and the largest percentages worked in the medical department (32.1%) and surgical department (27.5%; Table 1).

**TABLE 1.**
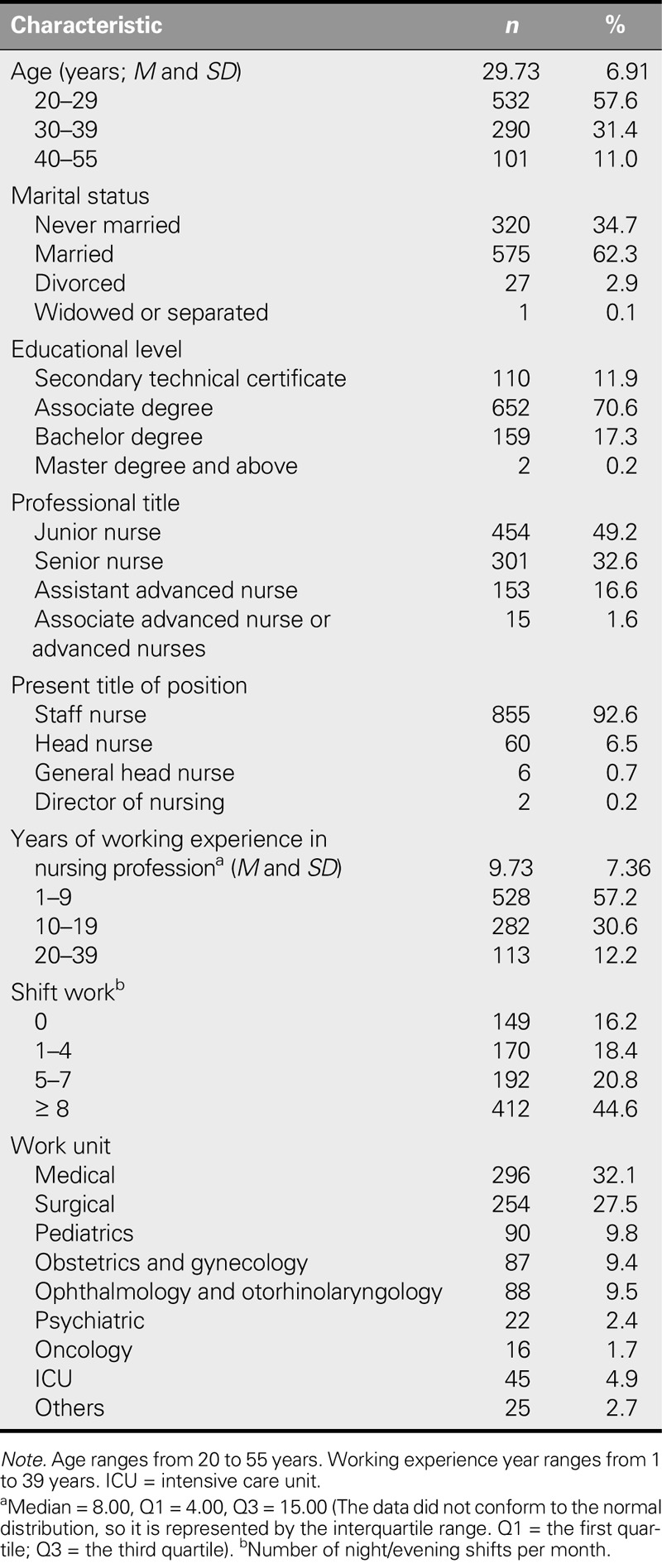
Demographic Characteristics of Participants (*N* = 923)

### Modification of the Original Model

**Figure 2.**
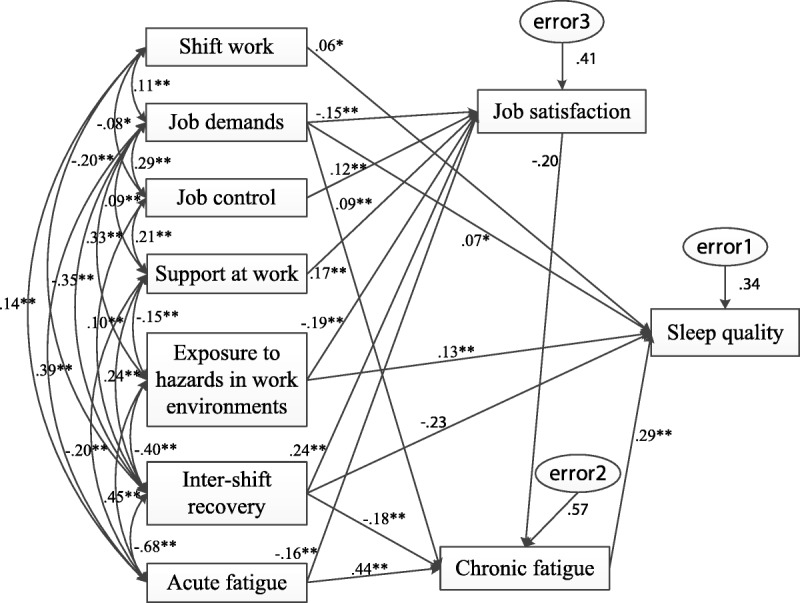
Standardized estimates of the final predicting model. *p < .05. **p < .01.

Pearson correlation analyses were initially used to examine the bivariate correlations among the variables (Table 2). The proposed associations between job control and sleep quality (*r* = .000, *p* = .991) failed to reach statistical significance. Thus, this pathway was ruled out in the following analyses, as per the prerequisite of conducting path analysis. Next, path analysis was performed to test, trim, and modify the hypothesized model. On the basis of theoretical reasoning and modification indices, three pathways (intershift recovery and job satisfaction/intershift recovery and sleep quality/acute fatigue and job satisfaction) were added to generate a much improved fit, whereas two nonsignificant pathways (support at work and sleep quality/job satisfaction and sleep quality) were removed to guarantee the parsimony of the final model. Multiple tests were conducted to compare the modified models, which yielded the best data-fit model (χ^2^ = 17.645, χ^2^/*df* = 1.961, RMSEA = .032, NFI = .994, CFI = .997, GFI = .996; Figure 2).

**TABLE 2.**
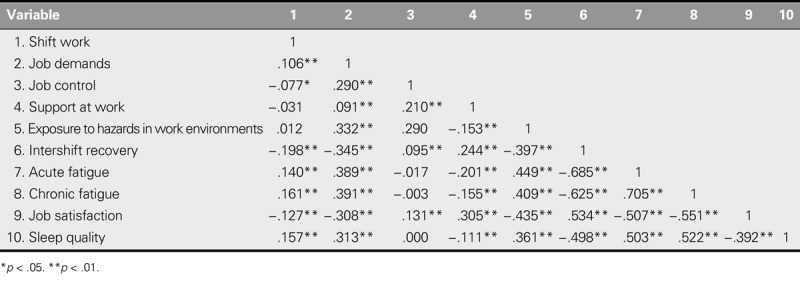
Correlation Matrix of Study Variables (*N* = 923)

### Predictors of Sleep Quality in Chinese Nurses

The final model accounted for 34.1% of the variance in the outcome of interest. Shift work, job demands, exposure to hazards in work environments, and chronic fatigue were found to affect sleep quality in Chinese nurses directly (total effect [TE] = .056, *p* = .041; TE = .108, *p* = .002; TE = .138, *p* = .002; and TE = .288, *p* = .002, respectively), which echoed our stated hypotheses. In addition, the mediating role of chronic fatigue was confirmed. Contrary to our initial expectation, intershift recovery was also found to exert a direct influence over sleep quality (TE = −.299, *p* = .004). Job satisfaction, the only indirect predictor of outcome that was identified (indirect effect [IE] = −.058, *p* = .001), should thus be regarded as a distal mediator. Contrary to our initial assumption, job control and support at work were found to have an indirect influence on sleep quality (IE = −.007, *p* = .001, and IE = −.010, *p* = .001, respectively), whereas, in line with our initial assumption, the effect of acute fatigue (IE = .136, *p* = .001) was found to be fully indirect. In addition, statistical significance was approached by all of the direct and indirect effects of sleep quality on the grounds of bootstrapping results (Table 3).

**TABLE 3.**
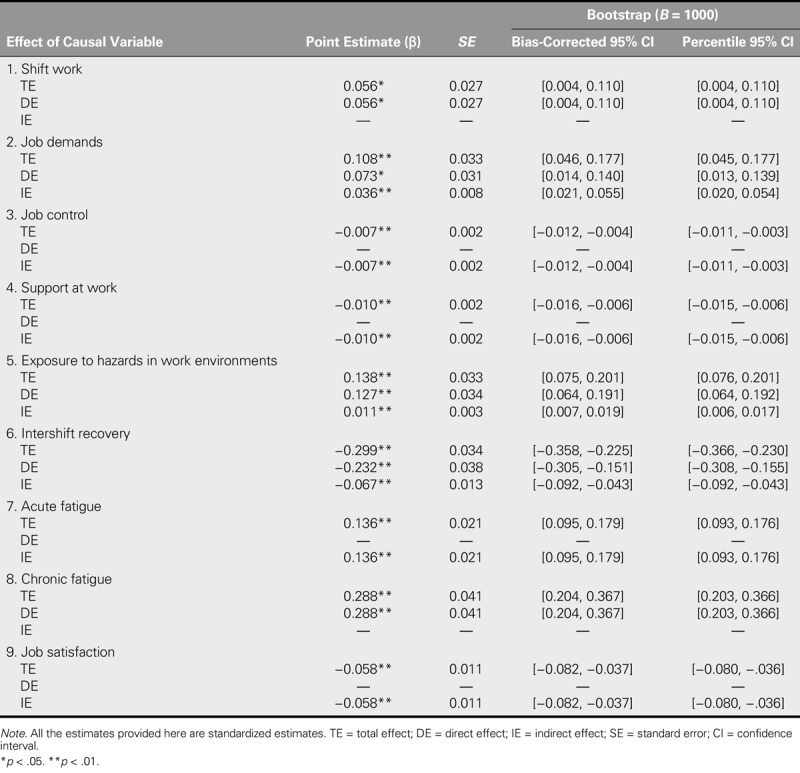
Direct, Indirect, and Total Effects of Causal Variables on the Outcome (*N* = 923)

## Discussion

Poor sleep quality in nurses is known not only to adversely affect their physical and mental health but also to cascade into other undesirable outcomes such as medical errors, degraded work performance, and even involvement in malpractice lawsuits (Eanes, 2015). This study prospectively examined the direct and indirect effects of modifiable work-related predictors of sleep quality in Chinese nurses using a path analysis model. The findings indicate that predictors included in the final model could explain 34.1% of the variance in sleep quality. According to Cohen et al. (2003), this result represents a medium-sized effect. Considering that only variables that were easily manipulated and related to work were proposed, 34.1% represents a relatively large proportion. These results indicate that countermeasures that target the factors in the developed model could play important roles in ameliorating poor sleep quality in Chinese nurses.

### Direct Predictors

The final model focuses on shift work, job demands, exposure to hazards in work environments, and chronic fatigue as direct predictors, which reflects both the original model and the findings of previous studies (Han et al., 2016; Hope, Øverland, Brun, & Matthiesen, 2010; Sandberg et al., 2016). More frequent night-shift work, implying more artificial light exposure and daytime sleep, negatively affects the normal circadian system, which regulates human sleep, suppresses daytime sleep, and promotes the secretion of melatonin (Korompeli et al., 2013). Enforced night-shift work may help explain why night-shift nurses face a high risk of poor sleep quality. There is scholarly consensus that more demanding work schedules increase the risk of sleep problems (Sandberg et al., 2016; Van Doorn et al., 2016). Excessive demands put employees at a greater risk of psychologically and physiologically exhaustion, which are known to impair normal sleep patterns. In addition, exposure to hazards in work environments, an intrinsic trait of certain professions, has been directly associated with sleep quality. Parallel evidence was found in a study of offshore personnel working on the Norwegian continental shelf (Hope et al., 2010). The nursing profession is unique in that it combines the regular threats of physical, mental, and chemical hazards with potential exposure to contaminated biological wastes. As a result, detrimental effects may occur spontaneously and be continuously aggravated for long periods afterward. Chronic fatigue, regarded as an illness or as a long-term condition, reduces overall health, of which sleep quality is a component. The mediating role of chronic fatigue between job satisfaction, job demands, intershift recovery, and acute fatigue and sleep quality was confirmed in this study, echoing the study of Fang et al. (2013).

Intershift recovery, in addition to its anticipated indirect effect, was found to act directly on sleep quality. Winwood et al. (2007) found that respondents who had a more fulfilling recovery between work shifts reported distinctly better sleep. Although humans are perceived to be well adapted to facing demanding environments, the capacity of the interrelated systems (e.g., physiological mechanisms) that underpin this resilience is not unlimited (Winwood et al., 2007). Persistent environmental stressors may destabilize the neuro-psycho-endocrine mechanism functions that regulate the stress response and result in maladaptive health outcomes, including sleep disturbance, if no timely, ameliorative interventions are implemented.

Taken together, systemic changes that address the fundamental issues underlying work arrangement, job intensity, and optimizing workplace conditions should be earnestly taken into account to offset the deleterious effects exerted by the direct, negative predictors of sleep quality. Likewise, sufficiently long nonworking periods should be included in work shift schedules by nursing supervisors to maximize the recovery of nurses from their late work-shift strain and to minimize cumulative fatigue.

### Indirect Predictors

Job satisfaction, job control, and support at work were found to act indirectly on sleep quality, which was inconsistent with our hypothesis. Perhaps, this discrepancy is because of differences between the sample in this study and those in the reference studies used. Job satisfaction, referring to how employees feel about their work, was concluded to be an indirect factor, exerting influence on nurse sleep quality through the function of chronic fatigue. Hence, this study interpreted job satisfaction as a distal mediator. The indirect effect of the proposed factors on sleep quality was found to be mediated first by job satisfaction and then chronic fatigue. Similar findings were presented by Kunzweiler et al. (2016), who explored the factors that influenced sleep quality in German nurses. Employees perceiving their job to be below the satisfactory threshold may exhibit declining interest or involvement and express feelings of being unvalued and depressed. Such a negative mental status tends to increase fatigue, undermine their overall fitness, and ultimately disturb sleep rhythm. Insufficient rewards and benefits as well as heavy workloads and responsibilities have been identified as the major job-dissatisfaction complaints among Chinese nurses (Chen & Fang, 2016). Job control and support at work were found to exert indirect, positive effects on sleep quality. The association between low control, as measured by repetitive work plus limited autonomy, and poor sleep quality was previously confirmed in a systematic review (Ruotsalainen et al., 2014). In addition, lacking support at work was found to be correlated with the increased reporting of sleep problems (Akerstedt et al., 2012). As illustrated by the demand–control model, job strain in workers that results from overwhelming demands and limited control coupled with little support in turn affects their physical and mental health (Sandberg et al., 2016).

Corresponding to our initial hypothesis, acute fatigue was identified as an indirect predictor of sleep quality. The progression from acute fatigue to chronic fatigue has been widely reported in other studies as well (Fang et al., 2013; Winwood et al., 2006). Only when promptly ameliorated can acute or transient fatigue become adaptive rather than stressful (Winwood et al., 2006).

To decrease emotional exhaustion and enhance sleep quality among Chinese nurses, organizational interventions should be implemented that target indirect as well as direct predictors. Implementing countermeasures to brighten nursing career prospects, enforcing empowerment strategies to improve decision latitude, and dissolving worksite apathy are all approaches that are recommended for implementation by nursing managers. Furthermore, recovery activities that prevent acute fatigue from progressing into chronic fatigue or even burnout should be actively provided.

### Limitations

The target population in this study included only nurses who were employed at general hospitals in urban areas of China. Therefore, the generalizability of findings may be limited. Because of the cross-sectional research design employed, inferences of causality cannot be drawn with certainty. Therefore, longitudinal or experimental designs are recommended in future studies of this topic. Moreover, all of the variables were measured using retrospective, self-report means. Thus, the influence of common method variance and self-report bias cannot be ruled out. Future research on this topic may include real-time assessments by using an experience sampling or a daily diary design. Moreover, path analysis is a structural equation model that is used in confirmatory rather than exploratory analysis. Thus, the thoroughness of the literature review and the construction of the original model impact the whole process significantly.

### Conclusions and Implications for Nursing

Sleep is necessary for good health, as individual wellness and occupational safety for nurses relate strongly to the quality of patient care. Therefore, joint efforts and systematic changes should be taken by policy makers, hospital administrators, and nursing managers to relieve sleep problems in nurses. Direct, modifiable work-related predictors as well as indirect predictors of sleep quality in Chinese nurses were revealed in this empirical study. Programs to effectively improve the significant adjustable factors to help nurses recognize and improve their sleep condition should be prioritized in organizational strategies.

Future research on improving quality of sleep in nursing personnel should focus on (a) appropriately arranging work shifts and human resource allocations, (b) utilizing effective tactics to alleviate stress and reverse fatigue, and (c) optimizing the physical and psychosocial work environment.
